# A modified squeeze equation for predicting the filling ratio of nanoimprint lithography

**DOI:** 10.1186/s40580-017-0108-z

**Published:** 2017-06-13

**Authors:** JiHyeong Ryu, Ho Lee, Sang-Ho Lee, HyungJun Lim, JaeJong Lee

**Affiliations:** 1 School of Mechanical Engineering, Kyungpook National University, Daegu, 702-701 Republic of Korea; 2 Division of Mechanical & Automotive Engineering, Wonkwang University, Iksan, 570-749 Republic of Korea; 3 Nano-Convergence and Mechanical Systems Research Division, Korea Institute of Machinery and Materials, Daejeon, 305-343 Republic of Korea; 4 Department of Nano Mechatronics, University of Science and Technology, Daejeon, 305-807 Republic of Korea

**Keywords:** Modified squeeze model, Filling ratio, Thermal nanoimprint lithography, Pressure variation rate

## Abstract

A numerical method using the modified squeeze model is proposed in this paper in order to overcome the limitation of the established squeeze equation and obtain filling ratios for nanoimprint lithography (NIL). Because the imprinting velocity is overestimated when the ratio of indenter width to polymer thickness is close to unity, the modified equation is critical. For verification, the numerical results are compared with the experimental data according to the various stamp geometries and pressure variation rates, for which a maximum difference of 10% is indicated. Based on these results, additional studies are conducted using the modified squeeze equation in order to obtain filling ratios according to the polymer thickness and temperature. The filling rates are enhanced through the increases in the temperature and the polymer thickness. The results demonstrate that the modified squeeze equation can be used to obtain and predict the filling ratio of sub-nanoscale NIL fabrication. It is expected that this study will assist in optimizing the experimental conditions and approaches for roll-to-roll NIL and step-and-flash NIL.

## Background

Nanoimprint lithography (NIL), which was proposed by Chou [1], is widely considered to achieve complicated structures [2, 3] for electronic devices. This method, however, has several technical issues to resolve before becoming more adept than conventional lithography methods. These issues include bubble defects, incomplete filling, stamp deformation, and residual layers. In order to solve these problems, numerical methods have been used to understand the polymer filling behaviors, which are crucial for achieving stable patterns and designing the imprinting conditions. Heyderman et al. investigated the filling characteristics of stamps during thermal NIL using poly(methyl methacrylate) (PMMA, M_w_ = 75 k). They observed two filling mechanisms for microcavities where a viscous flow moved into the cavity center from the edges and mounds were formed by capillary flow [4]. Scheer et al. analyzed the polymer filling behaviors according to the stamp geometry (indenter width, polymer thickness) and polymer properties (surface energy, viscosity, and molecular weight) based on hydrodynamic considerations in thermal NIL. They demonstrated that the squeeze theory could be used to compare different imprint situations [5]. King et al. simulated the polymer deformation according to the polymer thickness, cavity size, and hybrid asymmetric neighbor cavities [6]. They presented that the flow characteristics were defined according to the cavity width to polymer thickness ratio and polymer supply ratio: pipe, squeeze, and Stoke’s flow. Lee et al. investigated various polymer filling behaviors including the numerical methods according to the slip boundary condition, dynamic contact angle, pressure and temperature. It was shown that the polymer filling shape could be varied according to the pressure, temperature, and stamp geometry [7, 8]. Bonning et al. proposed a new simulation technique with a contact mechanical-based approach for thermal NIL. This method has the advantage of requiring 30–100 s for the NIL simulation. They demonstrated that the numerical results were in good agreement with the experimental data [9, 10].

In the present study, the modified squeeze model was developed in order to overcome the limitation of the established squeeze equation for NIL and used to predict the polymer filling behaviors and ratios. The numerical results were compared with the experimental data according to the various stamp geometries and pressure variation rates. Additional studies were conducted to obtain the filling ratio with various polymer thicknesses and temperatures using the modified squeeze equation. Experimental images were captured by scanning electron microscope (SEM) to obtain the filling shapes and filling ratios. It was found that simulation results using the modified equation were well in agreement with experimental data.

## Numerical method

### Overview

**Fig. 1:**
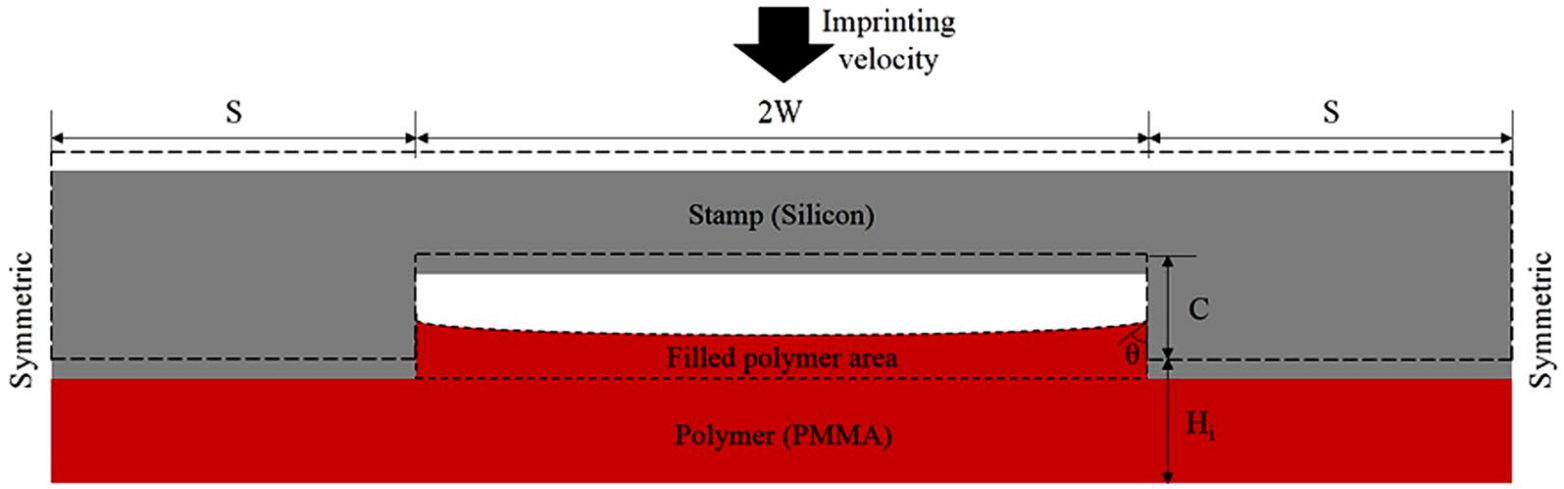
Simulation domain for NIL

The stamp was designed with line patterns to achieve a simple model using FLUENT software as shown in Fig. 1 and the its dimension was indicated in Table 1. As poly(methyl methacrylate) (PMMA, M_w_ = 75 kg/mol) was considered as a incompressible fluid under isothermal conditions, two governing equations [11], i.e. continuity and momentum equations, were used in the simulation. In order to consider the effect of the contact angles according to temperature, the surface energy of PMMA was referred from Fig. 1 in Ref. [12]. The contact angles between a substrate and a fluid were calculated using the followed equation:

**Table 1:** Dimensions of the silicon stamp for the NIL experiment

Parameters	Case 1	Case 2	Case 3
	Value (nm)	Value (nm)	Value (nm)
W (width)	600	1200	1800
S (indenter)	2200	2200	3400
C (depth)	250	250	250
H_i_ (height)	200, 300, 400	200, 300, 400	200, 300, 400

1 $$\uptheta = \arccos \left( {2\sqrt {\frac{{\upgamma_{\text{s}} }}{{\upgamma_{\text{l}} }}} - 1} \right)$$

where, γ_s_ and γ_l_ are the surface energy of the substrate and fluid, respectively. The surface energy of silicon with anti-sticking layer was obtained with 17.06 mN/m^2^. Table 2 showed the calculated contact angles using the variation of the surface energy with temperature. The simulations were carried out with same situation of the NIL experiments, where the pressure increased until 10 or 12.5 bar with pressure variation rate of 5.5, 10, 20, 50 bar/s and then, it was maintained for 1 s as shown in Fig. 2.

**Table 2:** Surface tension and contact angle according to the temperature

Temperature (K)	Surface tension (mN/m)	Contact angle (°)
428	33.70	65.0
433	33.35	64.5
438	33.00	64.0

**Fig. 2:**
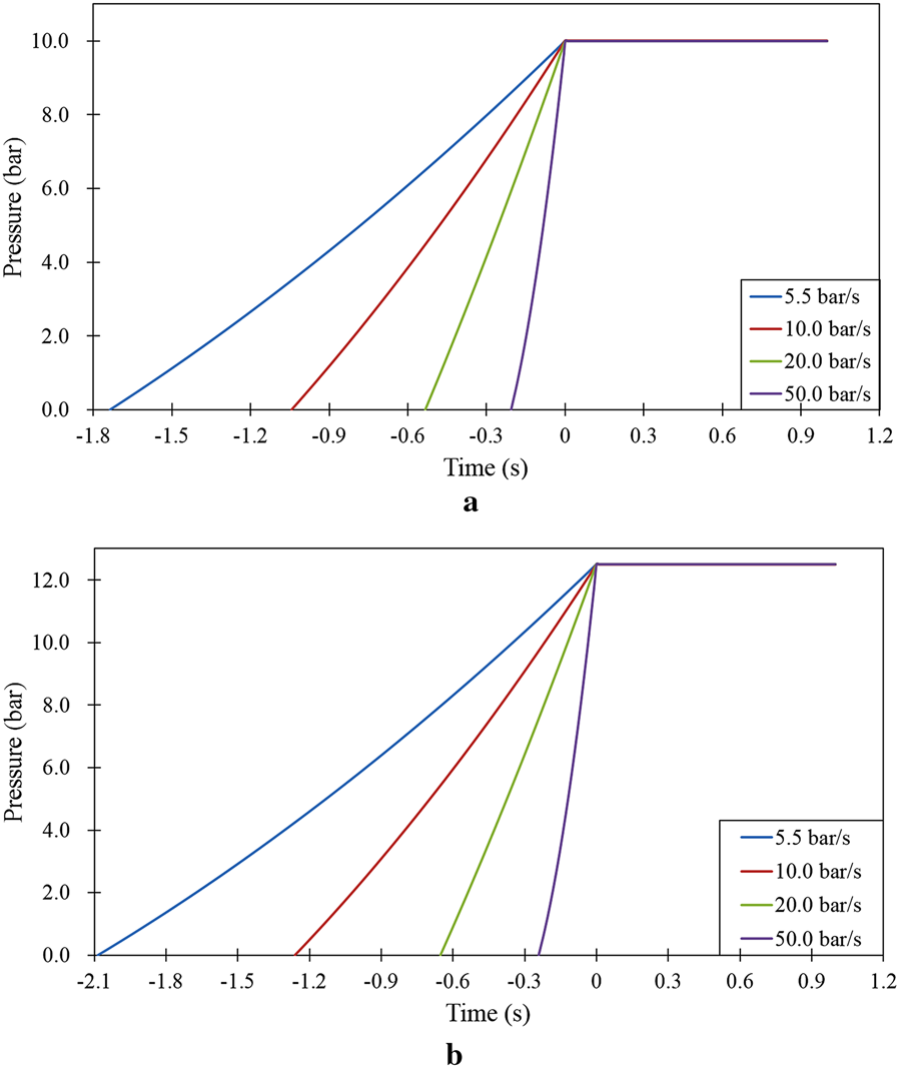
Pressure profiles with time until **a** 10 and **b** 12.5 bar with pressure variation rate of 5.5, 10, 20, 50 bar/s

### Polymer model

PMMA above the glass transition temperature (T_g_) is known as a shear thinning fluid which can be well described by the cross-WLF model presented by Walters et al. [13] as follows:

2 $$\upeta (\dot{\upgamma },{\text{T}}) = \frac{{\upeta_{{_{0} }} ({\text{T}})}}{{1 + (\upeta_{{_{0} }} ({\text{T}})\dot{\upgamma }/\uptau )^{{1 - {\text{n}}}} }}$$

3 $$\ln \left( {\frac{{\upeta_{0} ({\text{T}})}}{{\upeta_{0} ({\text{T}}_{0} )}}} \right) = \frac{{ - {\text{C}}_{1} ({\text{T}} - {\text{T}}_{0} )}}{{{\text{C}}_{2} + {\text{T}} - {\text{T}}_{0} }}$$

where $\dot{\gamma }$ and τ are the shear rate and critical stress level at the transition to shear thinning. *η*_0_(*T*) and *η*_0_ (*T*_0_) indicate the zero shear viscosity at the NIL temperature (*T*) and an arbitrary reference temperature (*T*_0_). *C*_1_ and *C*_2_ are the polymer specific constants, which can be determined from the fitted cross model using the results of the viscosity experiment. The parameters of the cross-WLF model for PMMA with 120 kg/mol, which indicates the shear thinning behavior, was presented in Table 3 [8]. Since the critical stress level is independent of molecular weight and depends only slightly on polymer chemical nature [14], all parameters can be used for the viscosity of PMMA.

**Table 3:** Parameters of cross-WLF model for PMMA experiment

Parameters	Value
C_1_	31.081
C_2_ (K)	51.6
T_0_ (K)	377.15
n	0.3973
τ (Pa)	35,607

The zero shear viscosity is proportional to the molecular weight (M_w_) below a critical molecular weight (M_c_). However, zero shear viscosity is dependent to the power of 3.4 of the molecular weight above the critical molecular weight. The critical molecular weight and is approximately 3 kg/mol in case of PMMA as presented by Torres [15]. Because the molecular weight of PMMA that we used is 75 kg/mol, the zero shear viscosity proportional to about the 3.4th power of the molecular weight can be used for this study. The zero shear viscosity was only extrapolated according to the molecular weight of PMMA. After the zero shear viscosity with the molecular weight was calculated, the viscosity values were followed by Eqs. (2) and (3).

## Experiment

**Fig. 3:**
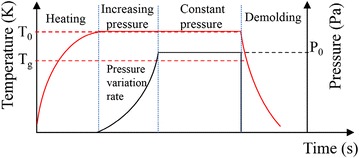
Procedure of NIL experiments and simulations with the pressure and temperature

The stamp with line patterns was fabricated via photolithography with the same dimensions as those used for the numerical method with the patterned area of 20 × 20 mm^2^ in the stamp size of 25 × 25 mm^2^. A fluorinated silane, tridecafluoro-1,1,2,2-tetrahydro-octyl-trichlorosilane (TFS, C8H4Cl3-F13Si) were treated in order to obtain anti-sticking layer for an easy demolding process before the NIL experiment. PMMA (Mr-I PMMA-75 k-300) with a thickness of 300 nm was coated onto a silicon wafer. The NIL process was conducted using laboratory-made equipment named as ANT4 in the Korea Institute of Machinery and Materials (KIMM) [16]. Figure 3 showed the procedure of NIL with imprinting pressure and temperature. A range of pressure variation rates of 5.5, 10, 20, and 50 bar/s, referred to as an ‘increasing pressure step’, were applied until 10 bar was reached at a temperature of 438 K using nanoimprint lithography to verify and compare with various numerical methods. In order to estimate the filling ratio with cavity size, we used the ratio of the cavity width to the tool width (dimensionless cavity ratio) and conducted NIL with the pressure variation rate of 10 bar/s under 12.5 bar at 438 K. After the pressures of 10 and 12.5 bar were reached, they were maintained for 1 s and given the name, ‘constant pressure step,’ in all NIL experiments.

Then, the filled cavity was rapidly cooled to 363 K in order to prevent creeping flow by thermal gradient following the pressing step. The experimental results were imaged using SEM to investigate the polymer filling ratios and behaviors for comparison with the numerical results. The filling ratio was approximated with SEM images and SolidWorks software, using the ratio of the filled polymer area to the cavity area. Experimental results were averaged in order to obtain the mean value, which is indicated by 86, 74, 67 and 64% with the variance of ±4% as the pressure variation rate increased from 5.5 to 50 bar/s. In case of the results of the dimensionless cavity size, the mean value was presented by 69, 81, and 90% with the variance of ±5%. The greatest difference between the means and error bars in the experimental results was approximately 8%.

## Results and discussions

### Modified imprinting velocity

**Fig. 4:**
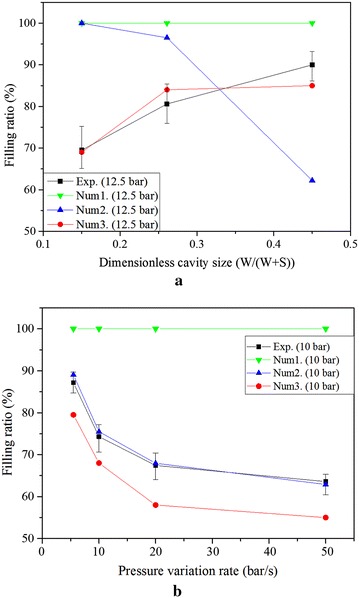
Filling ratios according to the **a** dimensionless cavity sizes and **b** pressure variation rates with various numerical equations at 438 K

Here, the stamp with line patterns descends into the polymer and then both of the materials are squeezed with a specific force. The line patterns allow the study of a mere cross section with a single period when the patterns are periodic. The imprinting velocity can be described using both the symmetric conditions boundary and the assumptions proposed by Stefan [17] as follows:

4 $${\text{V}}_{\text{NIL}} ({\text{t}}) = \frac{{{\text{P}}({\text{t}}) \cdot {\text{H}}({\text{t}})^{3} }}{{\upeta (\dot{\upgamma },{\text{T}}) \cdot (2{\text{S}})^{2} }}$$

5 $${\text{V}}_{\text{NIL}} ({\text{t}}) = \frac{{{\text{P}}({\text{t}}) \cdot {\text{H}}({\text{t}})^{3} }}{{\upeta (\dot{\upgamma },{\text{T}}) \cdot (2{\text{S}} + 2{\text{W}})^{2} }}$$

where *P*(*t*), *H*(*t*), and *η*(*t*) are the pressure, polymer thickness, and viscosity at each time step, respectively. The Eq. (5) was created with consideration of the area of the compressed air in the cavity to compare another equations. The established equations, i.e. Eq. (4), can be employed for the NIL condition for which the ratio of indenter width to the polymer thickness (polymer supply ratio) is more than 1. An imprinting velocity from classical squeeze model could be overestimated in the range of the ratio of indenter width of the stamp to polymer thickness that is close to 1 [6]. Thus, a compensated function is necessary to overcome this limitation:

6 $$\begin{aligned} {\text{V}}_{\text{NIL}} ({\text{t}}) &= \frac{{{\text{P}}({\text{t}}) \cdot {\text{H}}({\text{t}})^{3} }}{{\upeta (\dot{\upgamma },{\text{T}}) \cdot (2{\text{S}} + 2{\text{W}})^{2} }} \\ & \quad \times \left( {\frac{{2{\text{S}}}}{{2{\text{S}} + 2{\text{W}}}}} \right)\left( {\frac{\text{W}}{{({\text{H}}(0) + {\text{C}})}}} \right)^{\text{n}} \end{aligned}$$

where n is the index that can be changed according to the cavity size and ratio and a value of 1.7 was used for this simulation. The second term on the right is the weighted function by the ratio of indenter width to tool width. The last term is determined by the basis of the experimental results. The imprinting velocity was proportional to the cavity width, as shown in experimental results of Fig. 4a. *H*(*0*) + *C* was used as an inversely proportional function to prevent the overestimated effect of the initial polymer thickness and consider the flow resistance; this is described in Fig. 3 of Ref. [18]. We calculated the imprinting velocity under the quasi steady state every time step, where pressure, the viscosity, and polymer thickness were changed every time step. Because the pressure as the function of time developed the shear rate which was a parameter for the viscosity. The shear rate and viscosity can be calculated and updated every time step using the changed values of the imprinting velocity every iteration as below:

7 $$\begin{aligned} {\text{P}} &= {\text{P}}({\text{t}}),\quad\upeta ({\text{t}}) = \upeta (\dot{\upgamma },{\text{T}}),\\ {\text{H}}({\text{dt}}) &= {\text{V}}_{\text{NIL}} ({\text{t}})*{\text{dt}}, \\ {\text{H}}({\text{t}}) &= {\text{H}}(0) - {\text{H}}({\text{dt}})\end{aligned}$$

### Verification

**Fig. 5:**
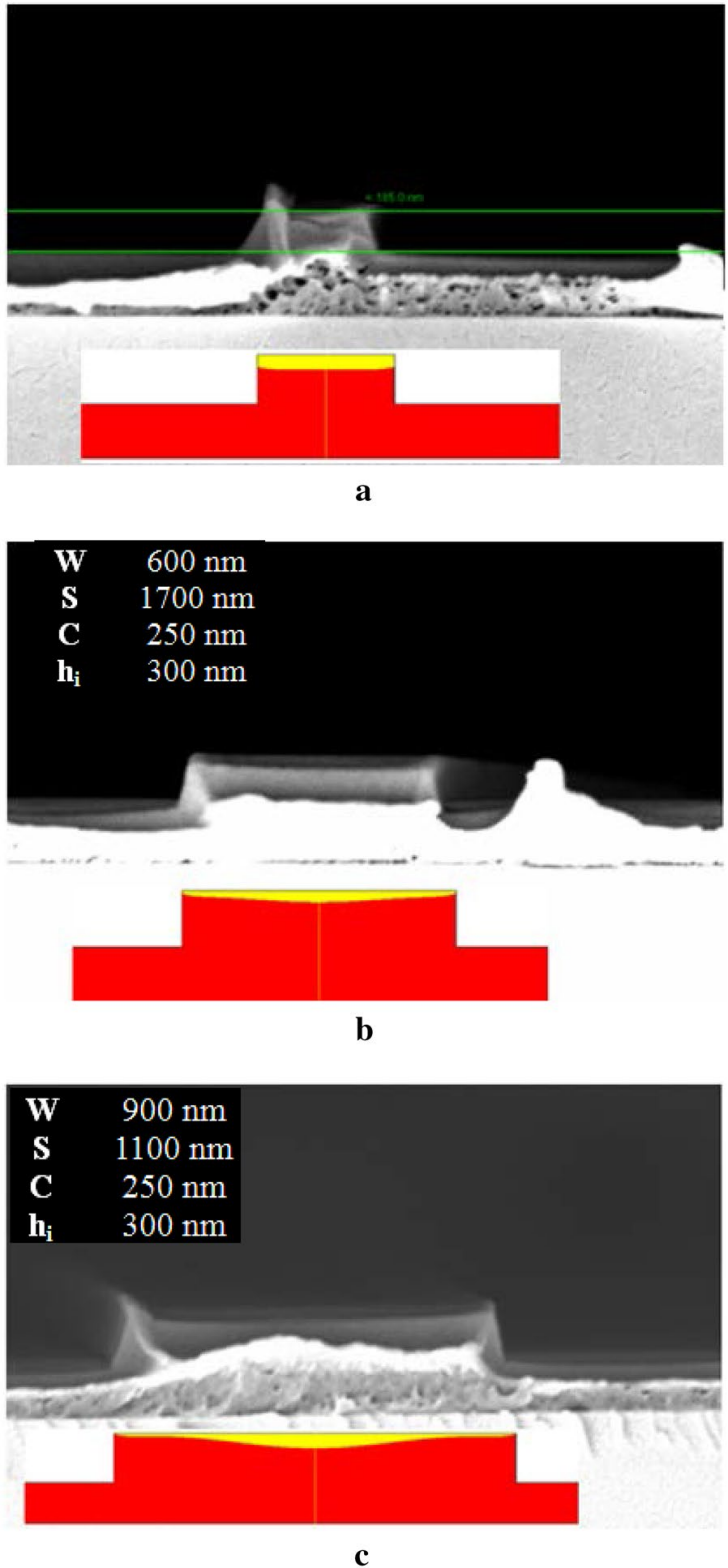
Numerical and experimental polymer filling behaviors according to different dimensionless cavity size of **a** 0.15, **b** 0.26, and **c** 0.45 with 10 bar/s at 12.5 bar in 438 K

Figure 4 presents the numerical and experimental filling ratio according to the ratio of cavity width to tool width [W/(S + W), dimensionless cavity size] and the pressure variation rate. SolidWorks was used for calculation of PMMA region and its values were divided by cavity area for the filling ratio from experiments. In case of numerical results, the FLUENT software can describe the occupied volume of PMMA in each meshes and calculate its values, using volume of fraction model. In experimental results, the filling ratio reduced with the increment of dimensionless cavity size due to growth of flow resistance. The similar tendency, which indicated the reduced filling ratio with the augment of the pressure variation rate, was observed. The numerical methods were carried out with same conditions of the NIL experiments. Num1, Num2, and Num3 indicated Eqs. (4), (5) and (6), respectively. In the case of Num1, PMMA perfectly filled in the cavity according to both the dimensionless cavity size and the pressure variation rate because of the height imprinting velocities, which were caused by too small an indenter width to estimate the filling ratios. Num2 was well matched with the results of the pressure variation rates. However, the filling ratios were inversely proportional to the dimensionless cavity size because the volume of the cavity became large compared with the similar calculated imprinting velocities. Num1 and Num2, which were the conventional squeeze model, were overestimated to predict the imprinting velocity for filling ratios at sub-nanoscale. Therefore the squeeze equation had to be modified like Num3 as the size of the patterns decreased. Num3 indicated that the maximum difference between numerical and experimental filling ratios were less than 10% and both results agreed well. The filling ratio increased and reduced with the increment of dimensionless cavity size and the augment of the pressure variation rate, respectively, as illustrated in Fig. 4. The phenomenon where the filling ratio increased with the increased dimensionless cavity size resulted from growth of flow resistance as the cavity width decreased. The numerical results was compared with experimental images which was carried on under the same condition of the simulation in order to understand the filling behaviors, as shown in Fig. 5. Both results indicated concave shapes due to the effect of capillary force and were in good agreement.

### Filling ratio with polymer thickness and temperature

**Fig. 6:**
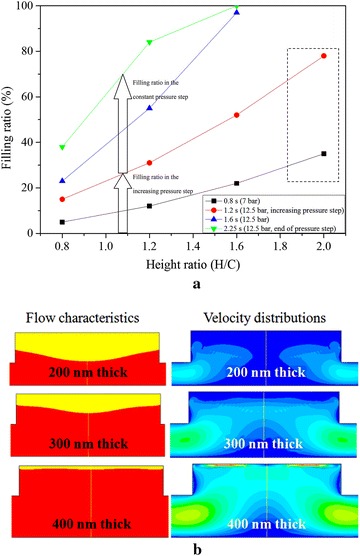
Variations of the **a** filling ratio with height ratios and **b** flow characteristics and velocity fields according to the polymer thickness. (Temperature = 438 K; pressure variation rate = 10 bar/s; and time step = 1.6 s)

Based on verifications, additional investigation using Num3 was conducted with various polymer thicknesses and temperatures. The values in the dotted line box in Fig. 6a indicated calculated filling ratios using extrapolation in order to understand the tendency. The simulation was conducted before the polymer perfectly filled in the cavity, indicating the simulation time.

**Fig. 7:**
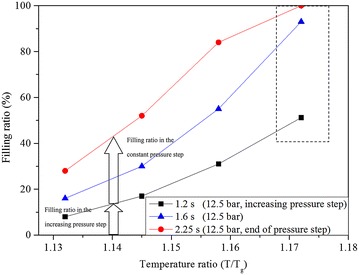
Filling ratio according to the temperature ratio. (Thickness = 300 nm; pressure variation rate = 10 bar/s; T_g_ = 378 K; and time step = 2.25 s)

Figure 6a illustrates the filling ratio increased with the ratio of polymer thickness to cavity depth (height ratio) at 438 K. The polymer thickness increased by 50, 100, and 150% from 200 nm, which means the thickness increases 100, 200, and 300 nm based on 200 nm resulted in increased filling ratios increased by 2.4, 4.2, and 6.5 times at 1.6 s, respectively. The filling ratios increased as much as the square of the increased times from the basis of polymer thickness. For example, when the polymer and base thicknesses are 300, and 200 nm, the filling ratio at 300 nm can be expected to grow as much as the square of 1.5 from it at 200 nm. Increases in the polymer thickness led to large filling ratios because the imprinting velocity was proportional to the polymer thickness. The polymer filling characteristics and velocity distributions are described at the end of the pressing step, which consists of an increasing pressure step and a constant pressure step, as depicted in Fig. 6b. The polymer filling behaviors changed according to the polymer thickness. As the polymer thickness decreased, a more concave shape was obtained. In contrast, a double peak shape was produced with a high imprinting velocity, which caused a dominant squeeze flow. The filling ratio with the temperature is presented in Fig. 7. The polymer viscosity is known as a function of the temperature and shear rate. The polymer viscosity decreased with increases in the temperature, which led to increases in the filling ratio and imprinting velocity. Table 4 presents the zero shear viscosity at each temperature. The zero shear viscosity decreased by 50, 75, and 87.5% from its value at 428 K, when the temperature increased by 278, 283, and 288 K and, correspondingly, the filling ratio increased by 1.9, 3.4, and 5.8 times, respectively, after a duration of 1.6 s, since the imprinting velocity was inversely proportional to the viscosity. The filling ratios can be predicted to be the decreased values to the power of 0.86 from basis of the zero shear viscosity. There were similar tendencies in both results with the polymer thickness and the temperature. The filling ratios at the constant pressure step were larger than those in the increasing pressure step because of the high averaged imprinting velocity in the constant pressure step (Note that results at increasing and constant pressure steps mean the filling ratios at the step which the pressure increases until 12.5 bar and which maintained 12.5 bar for 1 s) [19].

**Table 4:** Zero shear viscosity of PMMA at each temperature

Temperature ratio	Temperature (K)	Zero shear viscosity (×10^5^ Pa s)
1.13	428	2.4
1.14	433	1.2
1.15	438	0.6
1.17	443	0.3

## Conclusions

The numerical method using the modified squeeze model was proposed to predict filling ratios and surmount limitations of the conventional squeeze equation in this paper. Both the results of the modified method and the experiment had a maximum difference of 10%. Concave shapes were indicated in the results, which were well in agreement. The filling ratio increased with the increases of dimensionless cavity size, polymer thickness, and temperature. It was confirmed that the filling ratio was proportional to cavity width due to air resistance. The filling ratio increases by 2.4, 4.2, and 6.5 times were caused by the augmentation of the polymer thickness of 1.5, 2, and 2.5 times from 200 nm, respectively. In the case of the result of the temperature, the filling ratio augmented 1.9, 3.4, and 5.8 times as the zero shear viscosity reduced by 2, 4, and 8 times from the value found at 428 K. The results demonstrated that the modified squeeze equation can be used for sub-nanoscale NIL simulation and the filling ratio with various polymer thicknesses and temperatures can be predicted to be the square of the increased values from the base polymer thickness and the decreased values to the power of 0.86 from baseline zero shear viscosity, respectively. It is expected that this modified equation can be expanded to simulation at the nano-scale through adjusting the exponential index (n) and these studies will be helpful for creating adequate operating conditions and predicting filling ratios and times.
